# Does thrombo-aspiration still have a place in the treatment of myocardial infarction?

**DOI:** 10.1186/s12872-016-0291-6

**Published:** 2016-05-20

**Authors:** François Schiele, Fiona Ecarnot

**Affiliations:** EA3920, Department of Cardiology, University Hospital Jean Minjoz, 3 Boulevard Fleming, 25000 Besançon, France

**Keywords:** Thrombectomy, STEMI, Percutaneous coronary intervention

## Abstract

**Background:**

Thrombectomy for the treatment of ST elevation myocardial infarction (STEMI) is a simple and intuitive idea. In the 2000s, several studies evaluated the efficacy of thrombus aspiration and showed that thrombus aspiration led to improved myocardial perfusion, as assessed by a range of surrogate endpoints. These findings were confirmed by meta-analyses. However, the favorable results with thrombo-aspiration in STEMI were subsequently called into question by data indicating not only a lack of efficacy, but a risk of potentially deleterious complications.

**Discussion:**

We review here the scientific evidence in favor of, then subsequently against the utility of thrombo-aspiration in the setting of STEMI, and examine how such discordant findings come to be observed, e.g. technical problems, faulty study design, weak statistical power, or a true lack of efficacy of thrombus aspiration. We also consider what these conflicting results may mean for the future of this technique in the treatment of ST elevation myocardial infarction.

**Summary:**

Over the course of its development, significant evidence has cumulated both in favour of, and against thrombectomy for the treatment of ST elevation myocardial infarction. Overall, although its place among the therapeutic armamentarium for ST elevation myocardial infarction is now limited, it is likely that it will continue to be used to treat specific cases, after careful consideration of the limited success of our catheters at retrieving effective thrombus, the risk of stroke linked to the procedure, and the special attention that needs to be paid to avoid a risk of embolization during removal of thrombotic material.

## Background

Thrombectomy for the treatment of ST elevation myocardial infarction is a simple and intuitive idea. It doesn’t take much convincing that removing part or all of the thrombus that is blocking the artery before implanting a stent is beneficial, both in terms of the obstruction, and at the level of the microcirculation. The scientific evidence in support of this concept is based on several publications. Between 2002 and 2005, seven small-sized studies reported favorable results with thrombectomy, and four of these showed a significant difference in terms of arterial patency. A subsequent meta-analysis confirmed that thrombectomy prior to angioplasty improved coronary reperfusion [1]. Lastly, the TAPAS study [2], published in 2008 and including 1071 patients with STEMI, evaluated the efficacy of thrombus aspiration in terms of a surrogate criterion, namely myocardial blush grade. The results met the expectations of the cardiology community, showing that the thrombus aspiration group had better myocardial perfusion, regardless of whether assessed in terms of blush grade, ST resolution, or ST elevation persistence on the ECG. The clinical impact of these findings was reported 1 year later, with a spectacular 43 % reduction in all-cause mortality in the thrombus aspiration group [3]. Indeed, the editorialist in *The Lancet* heralded a victory over the no-reflow phenomenon [4], and – even though the reduction in mortality seems hardly credible in a study of 1000 STEMI patients – subsequent guidelines gave a grade IIA recommendation for routine thrombus aspiration [5].

### Efficacy and safety called into question

The earliest studies did not address the technical difficulties of manual thrombus aspiration. There was no mention of complications; effective thrombus retrieval was achieved in most cases, and there were few procedural failures. The magnitude of the reduction in mortality reported in the Thrombus Aspiration during primary percutaneous coronary intervention in Acute myocardial infarction Study(TAPAS) study also raised some eyebrows. Is it really possible to achieve such a gain? By way of comparison, the advent of stenting during balloon angioplasty did not lead to a reduction in mortality, and usually, studies with a sample size 10 times as large are required to show a reduction in mortality in STEMI. Two large, randomized studies ensued, aiming to evaluate clinical criteria, each including almost 10,000 patients, a randomized study associated with a registry study namely Thrombus Aspiration in ST elevation Myocardial Infarction in Scandinavia (TASTE) [6] and a large-scale international trial Thrombectomy with PCI versus PCI Alone in Patients with STEMI (TOTAL) [7]. The results in terms of myocardial reperfusion were less salient than in the TAPAS study, although they did tend in the same direction, and thrombectomy was shown to reduce distal emboli. However, the results in terms of clinical criteria were less encouraging, namely there was no benefit on mortality at 1 year in either study. As if the lack of benefit wasn’t disappointing enough, the very safety of thrombus aspiration was also called into question. A second meta-analysis of thrombus aspiration published in 2013 [8] reported a trend towards a higher risk of stroke with this technique. Furthermore, similar findings were reported in the 1-year follow-up of the TOTAL study, where a significant increase in the risk of stroke with thrombectomy was observed [9]. The accompanying editorial in the New England Journal of Medicine suggested “that the time has arrived to prepare a requiem for routine manual thrombectomy” [10], and again, subsequent guidelines took into account the new evidence, and lowered the level of recommendation for thrombus aspiration from IIA to IIB [11].

### Discussion

The fact that successive randomized studies produced disparate results is not unusual in cardiology, or in any field, for that matter. Naturally, the largest and most recent studies are the ones that stay foremost in the collective memory. The findings of TASTE and TOTAL strongly prompt the cardiological community to abandon the practice of thrombus aspiration once and for all, since it incurs a higher risk, for no clinical benefit. However, things are not always that simple in reality. We may recall that in 2006, for example, active stents were also called into serious doubt. So before we put the last nail in thrombectomy’s coffin, maybe a frank discussion of the efficacy and safety is warranted.

The risk of stroke seems to be the main adverse event associated with thrombus aspiration, and was brought to light most prominently in the TOTAL study. The proposed mechanism of stroke in this context is cerebral embolization of the thrombotic material extracted. This is a plausible theory, and if such were the case, it is conceivable that improvements in either the technique or the equipment, and the exercise of particular caution, could circumvent this risk. There is also the possibility of a difference in the risk of stroke occurring purely by chance. Indeed, the actual number of strokes was quite small. In the meta-analysis published in 2013, the actual difference in stroke between thrombus aspiration and PCI was 4 patients (13/1054 vs 17/1057) [8], and was 24 patients (60/5035 vs 36/5029) in the TOTAL study [7]. Additionally, the stroke may have been caused by other factors, not related to the procedure, as suggested by the fact that many of the strokes occurred more than 30 days after angioplasty.

Is the lack of efficacy related to technical problems? The results of thrombus aspiration observed in coronary artery disease are in stark contrast with the findings of 5 randomized studies in the context of stroke, all published in 2015 [12–16]. Why is thrombus extraction successful in stroke but not in STEMI? Given the vast experience of cardiologists in angioplasty procedures, and the large number of thrombectomy systems already tested in clinical practice, it is hard to believe that this problem could stem from poorly trained operators, or poorly designed endocoronary systems. It is more likely that cerebral thrombus, often of embolic origin, is easier to capture and retrieve than platelet-rich thrombus forming locally on an atherothrombotic plaque, as found in STEMI.

Or is the study design at fault? There are many examples of cases where the design of a study, the selection of patients, or the choice of endpoints had a major impact on the results. The fact that the efficacy of thrombus aspiration found in small trials could not be replicated in TASTE or TOTAL is not unexpected. Evidently, a reduction in mortality such as that observed in the TAPAS trial generates many hypotheses, but does not prove anything. However, doubts persist because of the non-selection of patients in these trials. In particular, TASTE and TOTAL investigated a strategy of systematic thrombus retrieval with randomization taking place before angiography, and thus, before it was known whether the quantity of thrombus justified thrombus retrieval or not. In routine practice, the operator decides to perform thrombus aspiration based on the size of the artery, the accessibility of the lesion, and the quantity of thrombus estimated by angiography. This important question will no doubt remain unanswered, since, in the TOTAL study, the artery was occluded in 65 % of cases, and per the study design, bailout thrombectomy was allowed if the initial PCI alone strategy failed, namely in case of persisting large thrombus after stent deployment [17]. Indeed, bailout thrombectomy was performed in 355 patients (7.1 %) of the PCI-alone group, and an additional 69 patients (1.4 %) crossed over from the PCI-alone to the thrombectomy group. Finally, subgroup analysis from TASTE and TOTAL does not help to identify the best candidates for thrombectomy, since the only significant interaction in the TASTE study concerned the efficacy of thrombus retrieval in women, and results seemed to be better in high-volume centres. In the TOTAL study, comparison of pre-specific subgroups according to TIMI thrombus grade (<3 vs ≥3, and <4 vs ≥4) did not show any significant difference in terms of the primary endpoint.

Some other design differences between the TOTAL and TASTE trials also deserve to be highlighted. Firstly, the data in TASTE come from a well-established Scandinavian multicenter registry, and therefore, the general population of which this group is representative may have a different mortality profile compared to the population represented by the sample in the TOTAL trial. Secondly, in TOTAL, events were adjudicated independently, whereas data were monitored as part of the usual registry validation in TASTE. Third, in the TOTAL trial, 74 % of patients had a thrombus grade 4–5, compared to only 32 % in TASTE. These discrepancies, amongst others, may contribute to the conflicting results observed in this area.

What about the endpoint ? It is hard to question the validity of all-cause mortality as an endpoint, as used in the TASTE study, although in the TOTAL trial, the primary endpoint was a composite of cardiovascular death, recurrent MI, cardiogenic shock, or new or worsening NYHA class IV heart failure occurring within 180 days. In the report of the 1-year follow-up from the TOTAL study [9], a meta-analysis is presented including all studies of this technique, with a pooled population of over 20,000 patients. The excess risk of stroke is statistically significant, but with relatively few events (85/9773 vs 59/9813, i.e. a difference of 27 strokes for 19,586 patients evaluated). Conversely, the difference in mortality is statistically non-significant (OR 0.90, 95 % CI 0.79–1.02), but numerically in favour of thrombus aspiration (452/10250 vs 503/10282). Thus, thrombus aspiration saves two lives, at the cost of incurring one stroke. It is regrettable that there was no comparison of the net clinical benefit in a population of selected patients after coronary angiography.

## Conclusions

So is there still room for thrombectomy? The interventional cardiologist would probably find it hard to say that there is no longer any room for thrombectomy, but it has to be acknowledged that its place is now considerably limited. Nonetheless, the principle of improving myocardial reperfusion is not called into question and it would be advisable to keep a few thrombus retrieval catheters in the stockroom for special cases. The indications need to be discussed, taking into account the limited success of our catheters at retrieving effective thrombus, the risk of stroke linked to the procedure, and the special attention that needs to be paid to avoid a risk of embolization during removal of thrombotic material.
